# Sex determination and gender expression: Reproductive investment in snails

**DOI:** 10.1002/mrd.22662

**Published:** 2017-02-27

**Authors:** Joris M. Koene

**Affiliations:** ^1^ Faculty of Earth and Life Sciences Department of Ecological Science Vrije Universiteit Amsterdam The Netherlands; ^2^ Terrestrial Zoology Naturalis Biodiversity Centre Leiden The Netherlands

## Abstract

Sex determination is generally seen as an issue of importance for separate‐sexed organisms; however, when considering other sexual systems, such as hermaphroditism, sex allocation is a less‐binary form of sex determination. As illustrated here, with examples from molluscs, this different vantage point can offer important evolutionary insights. After all, males and females produce only one type of gamete, whereas hermaphrodites produce both. In addition, sperm and accessory gland products are donated bidirectionally. For reciprocal mating, this is obvious since sperm are exchanged within one mating interaction; but even unilaterally mating species end up mating in both sexual roles, albeit not simultaneously. With this in mind, I highlight two factors that play an important role in how reproductive investment is divided in snails: First, the individual's motivation to preferentially donate rather than receive sperm (or vice versa) leads to flexible behavioral performance, and thereby investment, of either sex. Second, due to the presence of both sexual roles within the same individual, partners are potentially able to influence investment in both sexual functions of their partner to their own benefit. The latter has already led to novel insights into how accessory gland products may evolve. Moreover, the current evidence points towards different ways in which allocation to reproduction can be changed in simultaneous hermaphrodites. These often differ from the separate‐sexed situation, highlighting that comparison across different sexual systems may help identify commonalities and differences in physiological, and molecular mechanisms as well as evolutionary patterns. *Mol. Reprod. Dev. 84: 132–143, 2017. © 2016 Wiley Periodicals, Inc*.

## SEX DETERMINATION VERSUS SEXUAL SYSTEMS

Sex determination can generally be defined as the mechanism by which sexual characteristics develop. The sexual roles are classically divided into separate male and female individuals; in such gonochoristic animals, sex can be determined via sex chromosomes (where specific genes trigger a cascade of events that lead to the development of a male or female individual), via haplodiploidy (where ploidy levels determine sex), or even via the environment (e.g., where temperature triggers development into either sex). Beukeboom and Perrin (2014; updating Bull, 1983) recently reviewed the evolution of sex determination, providing a nice overview of all that is known about the usual model organisms. For example, they highlight the importance of the mammalian sex‐determining region present on the Y chromosome (SRY, also referred to as testis‐determining factor) and the ratio between X chromosomes, and autosomes in invertebrates (e.g., *Drosophila melanogaster* and *Caenorhabditis elegans*) for triggering the sex‐determination cascade that orchestrates development into a male or female. A recent evolutionary study of the mammalian protein‐coding gene repertoires present on the sex chromosome of the heterogametic sex (i.e., the Y or W chromosome) interestingly revealed that SRY is ancestral to most mammals, except for the egg‐laying ones (Cortez et al., 2014).

Extensive knowledge has also been gained on the physiological processes involved in sex determination and differentiation in gonochoristic animals—in particular, the events that follow the expression of the SRY protein, or the reciprocal wherein the absence of SRY generally leads to development of the “default” female sex (e.g., Kashimada and Koopman, 2010). Such knowledge is largely based on a number of model systems and human medical cases. Prominent examples contributing to our understanding of genetic sex determination are found in medical conditions resulting from either missing (Klinefelter's syndrome) or possessing extra copies (such as Turner's, Triple‐X, and Double‐Y syndrome) of sex chromosomes (e.g., Heard and Turner, 2011). Phenotypic problems are also known to arise from mutations in the genes coding for SRY, anti‐Müllerian hormone, testosterone receptor, or di‐hydrotestosterone converting enzyme (5α‐reductase). Such mutations and erroneous segregation of sex chromosomes often result in a difference between genetic and phenotypic sex (pseudohermaphrodites) due to a mismatch between the development of the primary and secondary sexual characteristics. In other words, the gender that is expressed behaviorally and phenotypically may not match the genetic gender, which can lead to all kinds of problems if not detected early enough during development (Heard and Turner, 2011). Another intriguing example is the case of gynandromorphy, which is reported to occur in many separate‐sexed species—for example, birds, insects, crustaceans (reviewed by Levin and Palmer, 2007). The underlying mechanism of this phenomenon has been worked out in chickens, where a specific mutation leads to development into a hen on one side of the lateral line and a rooster on the other (G1 gynandromorphy) (Zhao et al., 2010). Only the male side of the individual develops secondary male characteristics, such as a large wattle, large leg spur, and breast musculature.

The preceding examples illustrate the possible discrepancies between the genetic, gonadal, and phenotypic sex of an individual. They also highlight that most studies address sex determination in a separate‐sexed context, which leaves whole animal groups largely underexposed (Beukeboom and Perrin, 2014), resulting in large gaps in our general understanding of sex determination. When considering these other animal groups, one quickly encounters different types of sexual systems (i.e., asexual, parthenogenetic, selfing, hermaphroditic), which require a slightly different perspective to fully understand.

One of these lesser‐studied groups is the Mollusca, on which my focus will lie for the rest of this review. This highly diverse animal group offers an interesting opportunity to investigate multiple aspects of sex determination, sexual systems, and sex allocation. But before getting into the details, let me first explain these terms (see also Box 1). Here, I define a sexual system as the way in which an organism expresses whether it is male, female, or both at the same time or in sequence—respectively, simultaneous or sequential hermaphrodites. As the resulting behavioral expression is often flexible, I refer to this outcome as gender expression. Obviously, the definition of sex determination becomes difficult to apply in such a context since both sexes are expressed in the same individual; this can be resolved by talking about “sex allocation” rather than sex determination. Sex allocation is used to indicate the division of reproductive resources between male and female reproduction (Charnov, 1979; Schärer, 2009). Although this may superficially seem rather different from how the term is used in separate‐sexed organisms, the difference essentially lies in where energy is invested (see also Schärer, 2009; Schärer and Ramm, 2016): In separate‐sexed organisms, sex allocation indicates the decision about the offspring's sex ratio—that is, the relative amount of energy that is invested in the production of sons and daughters—whereas in hermaphrodites, sex allocation decisions are made over the timing of sex change (in sequential hermaphrodites), and the division of resources between the individual's male and female function (such as gamete production and choice of mating role).

**Table:** 

**Box 1** Overview of the Definitions of Terms Used Throughout This Review	
Term	Definition
Allohormone	Substance that is transferred from one individual to another free‐living member of the same species. It can induce a direct physiological response, bypassing sensory organs.
Gender expression	The behavioural outcome of the way in which an organism expresses whether it is male, female, or both at the same time or in sequence.
Protandry	The male sexual function of the simultaneous hermaphrodite is engaged before the female function.
Reciprocal mating	Both mating partners perform both sexual roles at the same time. As a result, gametes (either eggs or sperm) are exchanged during a single mating interaction.
Role alternation	The swapping of sexual roles once a first (primary) mating has taken place between two mating partners.
Separate‐sexed	Organism possessing exclusively either functional male or female organs during its lifetime.
Sequential hermaphrodite	Organism possessing functional male and female organs in sequence (over time), meaning that they go through sex change at some point in their lifetime.
Sex allocation	The division of reproductive resources over male and female reproduction.
Sex determination	The mechanism by which sexual characteristics develop.
Sexual dimorphism	Differences in external appearance between the sexes, particularly in separate‐sexed organisms.
Sexual function	Term used to specify whether resources are invested into the male or female side of a hermaphrodite (i.e., the male function and female function, respectively).
Sexual system	The way in which an organism expresses whether it is male, female, or both at the same time or in sequence.
Simultaneous hermaphrodite	Organism possessing functional male and female organs at the same time (once mature).
Unilateral mating	Each mating partner performs only one sexual role at a same time. As a result, gametes (either eggs or sperm) are donated in one direction.

## BEHAVIORAL GENDER EXPRESSION AND HERMAPHRODITISM

Molluscs comprise the second most speciose group of animals, after arthropods. This class of animals contains an extensive variety of forms—ranging from slugs and snails, via bivalves, to cephalopods—and includes many marine, freshwater, and terrestrial species. All possible forms of sexual systems can be found, including separate sexes, asexuality, parthenogenesis, selfing, sequential, or simultaneous hermaphroditism (e.g., Michiels, 1998; Anthes, 2010; Auld and Jarne, 2016). Surprisingly little is known about the genetic basis of sex determination in these animals (e.g., Collin, 2013; Auld and Jarne, 2016). One given is that separate‐sexed mollusc species may utilize any of the known sex determination systems: XY, ZW, or XO (Nakamura, 1986; Barsiene et al., 2000; reviewed in Yusa, 2007); however, this knowledge is limited to a handful of model organisms, including several separate‐sexed bivalves and snails, such as nerites, apple snails, and river snails (Yusa, 2007). Hence, this field of research still offers plenty of opportunity for investigating and comparing sex determination in molluscan species with different sexual systems (Yusa, 2007; Auld and Jarne, 2016).

Sexual dimorphism in separate‐sexed molluscs can be detected in the shape of the shell and/or in the external appearance and body size (reviewed in Yusa, 2007; Collin, 2013). The same holds true for sequential hermaphrodites. A well‐known example of this dimorphism is found in slipper snails (genus *Crepidula*): When small (and young) individuals settle on larger (older) female individuals, the former mature as males; conversely, solitary settlers become females. The small males will eventually change sex, particularly when the settled‐upon female dies; but also, when individuals are stacked, the ones near the base of the stack are females (Hoagland, 1978; Yusa, 2007; Collin, 1995, 2013). In contrast, sexual dimorphism in the form of external morphological sex differences are absent in simultaneous hermaphrodites, by definition. Indeed, body size is clearly a trait of the whole organism and cannot be attributed to only one sexual function, even though there is often a positive relationship with egg production (Nakadera and Koene, 2013); moreover, body size is often confounded by age (Nakadera et al., 2015). To the best of my knowledge, no study on hermaphroditic molluscs has properly evaluated whether there are more subtle differences or traits that signal an individuals’ sexual function allocations. This topic is worth investigating, although one has to keep in mind that such traits may show phenotypic plasticity in response to environmental factors unrelated to sex allocation (e.g., Schärer, 2009; Nakadera and Koene 2013; Janicke and Chapuis, 2016).

For the remainder of this review, I will focus on hermaphroditism in molluscs. As observed for many organisms (Michiels, 1998; Schärer, 2009; Anthes, 2010; Koene, 2012; also see articles in this issue), contemplation of this sexual system allows for a different vantage point to address outstanding questions that cannot be tackled in separate‐sexed animals. When investigating and thinking about hermaphrodites, several important issues should be considered:

1. Hermaphrodites possess both sexual functions, either sequentially or simultaneously. It is also possible that they start out as one sex, and subsequently become simultaneous hermaphrodites (usually protandry, starting with male function before acquiring female function). So, hermaphrodites generally produce eggs and sperm, either in overlapping or sequential periods, over their lifetime (e.g., Ghiselin, 1969).
2. An individual can donate and receive sperm (e.g., Michiels, 1998; Anthes, 2010). This reciprocity can occur simultaneously within one mating interaction (direct exchange), sequentially within one or more mating interactions (indirect exchange via unilateral mating), or over the animal's lifetime (sequential hermaphroditism). The resulting exchange means that, on average, whatever is done to an individual's partner during a mating (donating nutrients or inflicting harm) could come back to that individual later on, given that mating will occur again in the complementary role (i.e., “what you give is what you get”).
3. In separate‐sexed animals, males generally bestow sperm as well as other substances, among which are many accessory gland products, to females. As a consequence, males can affect female reproductive physiology (Chapman and Davies, 2004; Ravi Ram and Wolfner, 2007). In contrast, simultaneously hermaphroditic sperm donors can transfer substances that potentially target either or both of the sexual functions of its partner.

**Figure 1:**
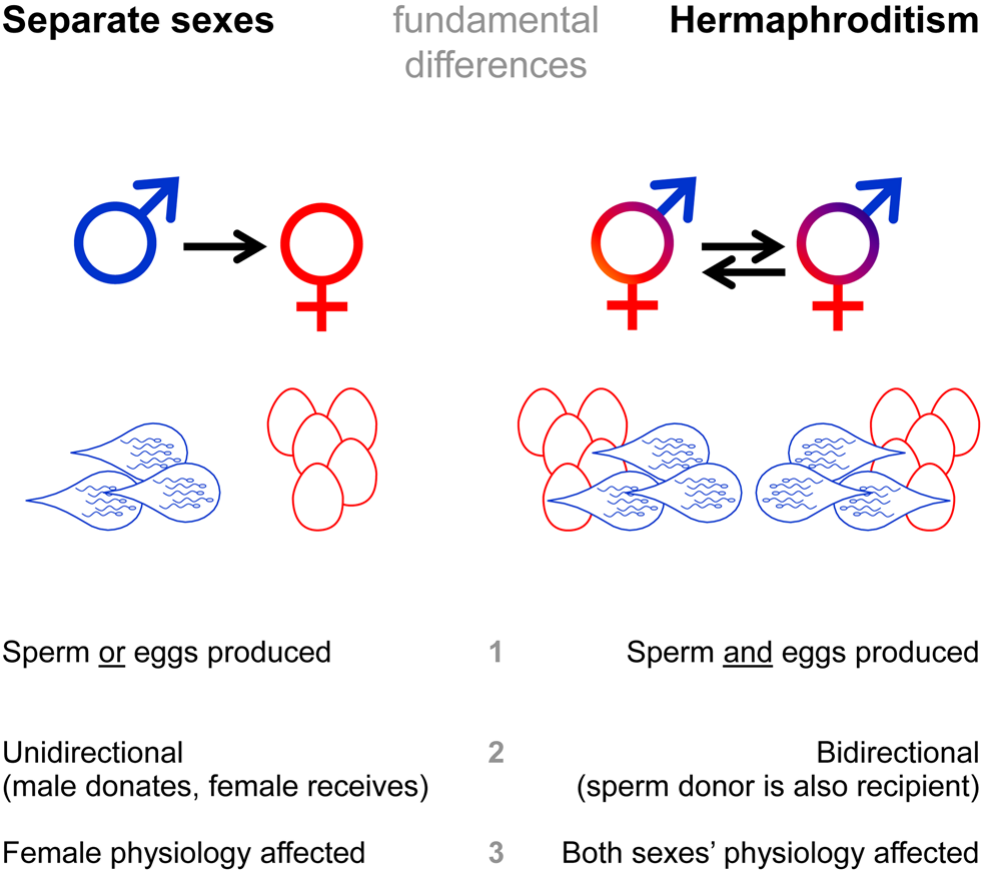
Illustration of the difference between separate‐sexed and hermaphroditic animals. In separate‐sexed animals, sperm and eggs are produced in different individuals whereas they are produced within the same hermaphroditic individual. As a result, gametes (usually sperm) are actively transferred between mating partners. This exchange can be direct (reciprocal mating) or indirect (unilateral mating). In either case, such interactions are often accompanied by the transfer of accessory gland products, as in separate‐sexed species, here indicated as a drop outlined around the spermatozoa (but note that sperm and accessory gland proteins can also be transferred separately; see text). One important difference between their function in separate‐sexed versus hermaphroditic individuals is that in the latter accessory gland proteins can influence sex allocation by targeting the male as well as the female physiology of the recipient (mating partner).

These considerations are expressed schematically in Figure 1, and I will illustrate this with several specific examples in the following sections. Before doing so, however, I will briefly revisit sex allocation theory, as this is important for what follows.

## SEX ALLOCATION AND HERMAPHRODITES

The topic of sex allocation has been extensively and thoroughly reviewed on several occasions (e.g., Charnov, 1982; Schärer, 2009), so I will only point out the essence of sex allocation theory before moving on to specific examples. The allocation of resources to the two different sexual roles of a hermaphrodite should ideally be done in such a way that they can maximize their reproductive success. In many sequential hermaphrodites, the decision to change sex can indeed be predicted based on whether there is more to gain by remaining in one sexual function or by changing to the other. A recent study asked this for slipper snails by basing the prediction of sex change on the reproductive success gained via either function (Broquet et al., 2015), using the size‐advantage model (Ghiselin, 1969; Warner, 1988; Munday et al., 2006). Broquet and coworkers were able to confirm that small individuals were better off as males since larger individuals had a fitness advantage as females, thus making protandry adaptive (Broquet et al., 2015).

In simultaneous hermaphrodites, sex allocation predictions are based on the theoretical framework of Charnov (1982), including recent extensions (e.g., Schärer, 2009; Schärer and Pen, 2013). Charnov pointed out that at least one of the sexual functions needs to show diminishing results in order for simultaneous hermaphroditism to be a stable reproductive strategy (Charnov, 1979). The female‐gain curves of hermaphrodites generally do not seem to saturate (Schärer and Pen, 2013), whereas the male‐fitness‐gain curves can show diminishing returns rather than being linear, as might be expected based on Bateman's principle (Bateman, 1948; Charnov, 1982). Thus, models predict that sex allocation will be female‐biased, which seems to be the general empirical pattern for hermaphrodites (Schärer, 2009; see also Schärer and Pen, 2013). These models hinge on data about the strength of (local) sperm competition, and assume that equal sex allocation occurs primarily in situations with large mating groups (i.e., polyandry). So from the perspective of male reproductive success, investment in sperm (and the transfer of accessory gland products) is optimal when mating frequency is high and sperm competition is strong. As we will see, there may be ways to assess if this leads to the available reproductive resources being equally divided over investment in male and female reproduction.

## MEASURING GENDER EXPRESSION AND SEX ALLOCATION

Two important factors need to be considered for assessing sex allocation and the behavioral expression of gender in hermaphroditic snails. On the one hand, individuals will try to maximize the sum of their male and female reproductive success (see also Schärer et al., 2015), leading to situations where individuals are more motivated to perform one sexual role than the other (e.g., Koene and Ter Maat, 2005, 2007; Nakadera et al., 2015). This internal motivation, explained below, requires flexible allocation of resources to either sex function. On the other hand, this motivation interacts with a second important factor: the effects caused by accessory gland products that are transferred during courtship and/or copulation. The following examples are used to explain how these factors affect gender expression and sex allocation.

### Motivational State for Gender Expression

The internal motivational state of the animal plays an important role in (behavioral) investment decisions, which may not be surprising if one considers that many snails and slugs invest a significant amount of their time and energy into reproductive behavior (e.g., Baur, 1998). Many environmental factors—such as density, temperature, food availability, predators, parasites, etc.—also influence whether mating will take place or not (e.g., Nakadera and Koene, 2013). Yet even after excluding such factors, differences in motivational state can still be observed. There are two main reasons for this: the profitability to mate (in one of the sexual roles) and the fact that the time since last mating—as male, female, or both—can temporarily sexually (de)motivate the individual.

Among the studied aspects related to the profitability to mate, sexual isolation is one of the main factors causing variation in motivation. Increased eagerness to mate after a period of sexual isolation seems to be a common phenomenon in a range of simultaneously hermaphroditic gastropods, including *Aplysia fasciata* (Ziv et al., 1989) and *Helix aspersa* (Adamo and Chase, 1990). Isolation is therefore often used experimentally to increase the likelihood of mating (reviewed in Koene and Ter Maat, 2005), and thus to observe a species' copulation strategy, as in the case of the hermaphrodites *Stagnicola elodes* and *Biomphalaria glabrata* (Rudolph, 1979a; Vernon and Taylor, 1996). These animals generally mate unilaterally, so it is important to consider which role drives their decision to mate. Such decisions also impact reciprocally mating species, but because they perform both sexual roles at the same time, disentangling male from female motivation is much more difficult (e.g., Michiels, 1998; Koene and Ter Maat, 2005).

The decision to mate has been investigated in detail in the great pond snail, *Lymnaea stagnalis*, which becomes more motivated after sexual isolation. The motivation to mate in the male role seems to dominate in this species, and can be clearly observed because of its unilateral mating strategy. Early studies showed that individuals who were sexually isolated for a longer period of time than their partner were more likely to act as sperm donors (Van Duivenboden and Ter Mat, 1985). When both individuals were isolated for the same number of days, and thus equally motivated to donate sperm, role alternation was observed, meaning that both individuals of the mating pair had the opportunity to donate and receive sperm (Koene and Ter Maat, 2005). In such instances, the individual that inseminated first is generally referred to as the primary donor and the partner as the secondary donor (Nakadera et al., 2014, 2015). To ensure that it also gets to inseminate, the secondary donor tends to assume a typical mating posture in which it holds onto the shell of its partner, ready to mount, even before the primary insemination finishes (Koene and Ter Maat, 2005). Observations on other freshwater snail species also indicate that such role alternations occur following sexual isolated (e.g., *Physa heterostropha pomilia* (Wethington and Dillon, 1996) but not during spontaneous copulations between non‐isolated snails [e.g., *Bulinus globosus* (Rudolph, 1979b); *L. stagnalis* (Van Duivenboden and Ter Maat, 1988); *Physa heterostropha pomilia* (Wethington and Dillon, 1996)].

Neurobiological work informs us about the underlying mechanism determining this motivational state. Studies on *L. stagnalis* revealed that an individual's “drive” to perform the male role is largely determined by the availability of seminal fluid in its prostate gland, where accessory gland proteins are produced. As it turns out, sexual isolation gives the animal time to fully replenish the seminal fluid cache that was largely spent in previous matings in the male role—a change can be confirmed by measuring the gland's weight (De Boer et al., 1997). The animal receives information about the fill state of its prostate gland, as demonstrated by electrophysiological experiments showing that increases in gland size are detected by the central nervous system via a small branch of the penial nerve, the np1 (De Boer et al., 1997; reviewed in Koene, 2010, 2016). This nerve branch feeds into the different regions within the central nervous system that are known to be involved in male mating behavior (reviewed in Jarne et al., 2010; Koene, 2010; El Filali et al., 2015).

Understanding how information is relayed to the central nervous system also makes it possible to experimentally interfere. When the np1 nerve is lesioned via a microsurgical procedure, animals no longer mate in the male role and are behaviorally completely feminized (De Boer et al., 1997). Such animals instead double the amount of eggs they lay compared to their hermaphroditic counterparts (De Visser et al., 1994; reanalyzed in Koene et al., 2009). This finding was initially interpreted as evidence that the resources gained by no longer investing in the transfer of ejaculates were reallocated to the female function, that is, egg production. (These snails are simultaneous hermaphrodites, so they also produce eggs; egg production is here used as the “currency” to measure investment.) While that experiment demonstrated that the investment in the two sexual functions is roughly equal for this species, and that the freed‐up male reproductive resources can be reallocated to female reproduction (Charnov, 1982), it did not fully disentangle investment in the male versus female role. To do so, a follow‐up experiment also included treatments in which individuals were restricted to mating in one sexual role, as opposed to being able to mate in both roles or none at all (Hoffer et al., 2010). That study revealed that single‐sex copulants, which were only able to mate as either males or female, each produced as many eggs as hermaphroditic (reciprocal) copulants. Thus, mating as only male or only female uses up an equal portion of the reproductive budget, implying that the amount of energy invested in each sexual role is equal (although we will see, in the next section, that the reduction in egg laying in the female‐only group also has a different cause).

The above illustrates how knowledge about the underlying physiological mechanism can provide insight into energy allocation between the two sexual functions as well as explanations for behavioral decisions about gender expression. The observation that the energy put into the male function equals that of the female function also aligns with behavioral work showing that offering an unfamiliar partner can also increase male motivation (Koene and Ter Maat, 2007). Such increased motivation to mate with a novel partner, known as the Coolidge effect (Wilson et al., 1963), is in line with the idea that animals will allocate their ejaculates strategically if the transfer and/or production is energetically costly (Dewsbury, 1982; Pizzari et al., 2003; Koene and Ter Maat, 2007), thus corroborating the measurements of investment in *L. stagnalis* explained above. This same effect was hypothesized to occur in the sea hare, *A. fasciata* (Ziv et al., 1989, but never experimentally tested), but was not found in the Ramshorn freshwater snail, *B. glabrata* (Häderer et al., 2009) and the sea slug, *Chelidonura sandrana* (Werminghausen et al., 2013).

### Relationship Between Accessory Gland Products and Sex Allocation

A crucial role for accessory gland products is supported by the observed male behavior of *L. stagnalis*, which seems to be mainly regulated by the availability of seminal fluid in the accessory prostate gland and not sperm in the seminal vesicles (although this organ is also generally innervated—for example, the land snail *Cornu aspersum* (Geoffroy et al., 2005). Indeed, seminal fluid components, produced in accessory glands, play a major role in post‐copulatory sexual selection processes in many animals (e.g., Arnqvist and Rowe, 2005; Perry et al., 2013). This section focuses on the different effects of specific accessory gland products in relation to gender expression and possibly sex allocation in two different molluscan model species, *C. aspersum* and *L. stagnalis*, not only because these are best investigated in this respect but also because they exhibit two very different ways of transferring accessory gland products: respectively, separated from the sperm via hypodermic injection, or along with the sperm via the ejaculate (Zizzari et al., 2014).

The brown garden snail *C. aspersum* is the model species used for much of the work related to the transfer of accessory gland products via hypodermic injection in simultaneous hermaphrodites (Koene and Schulenburg, 2005; Lodi and Koene, 2016a,2016b). This species mates simultaneously reciprocally, with sperm packages (called spermatophores) being exchanged during a single mating interaction. During courtship, and prior to spermatophore transfer, each individual attempts to stab (rather violently) a so‐called love dart through the body wall of its mating partner. In doing so, the love dart injects the mucus with which it is coated into the partner's haemolymph. This mucus is produced by an accessory organ called the digitiform gland that is associated with the dart‐sac. The mucus causes conformational changes to the part of the female reproductive system that receives the spermatophore, resulting in altered spermatophore uptake and delayed sperm digestion (Koene and Chase 1998a), thereby increasing sperm storage (Rogers and Chase, 2001), and ultimately paternity of the successful dart user (Landolfa et al., 2001; Rogers and Chase, 2002; Chase and Blanchard, 2006).

The study by Koene and Chase (1998a; see also Lodi and Koene, 2016a,2016b) has so far been the only research to use control mucus extracts, reporting that mucus of the pedal gland (which produces the mucus trail) evoked a rather similar response. This indicates that the active substance is either a general constituent of mucus or that the two extracts cause a similar effect via different mechanisms. Obviously, it seems rather unlikely that the pedal gland products enter into the haemolymph via dart shooting (although this may occur with mucus present on the skin). Irrespective of whether or not a mucus component from other sources evokes a similar effect, this would only indicate that the active component is possibly also used in a different context than dart shooting—which is not unusual for accessory gland products (e.g., Yi and Gillott, 2000). The latter scenario is strengthened by the identification of the digitiform gland component that induces one of the muscular contractions in the spermatophore receiving organ: the active peptide turns out to resemble buccalin, a known modulator of which different forms are used to change muscle contractions in freshwater and marine molluscs (Stewart et al., 2016). This particular peptide was named love‐dart allohormone (LDA), and is found in both *C. aspersum* and *Theba pisana*, the two species investigated by Stewart et al. (2016). The latter agrees with recent work showing that the effects of dart mucus are evolutionarily conserved within a number of dart‐bearing species (Kimura et al., 2014; Lodi and Koene, 2016a,2016b).

While it remains to be demonstrated if LDA is responsible for the increase in sperm storage after successful dart shooting, other effects have also been recently reported. For example, *Euhadra quaesita* (Bradybaenidae) snails stabbed during mating experienced a longer remating interval compared to unstabbed individuals (Kimura et al., 2013) ‐ again, not an unusual effect of accessory gland products (e.g., Scott, 1986; Liu and Kubli, 2003; Himuro and Fujisaki, 2008). If dart receipt also induces ovulation, as suggested by Kimura et al. (2013), its action might influence sex allocation of the partner being targeted; but note that Koene and Chase (1998b) reported that receiving a dart did not affect oviposition in *C. aspersum*, indicating that this hypothesis needs further investigation.

Accessory gland products can also be transferred along with the sperm in the ejaculate—in which case they may be referred to as seminal fluid products or proteins (Perry et al., 2013), although there is no reason to assume that they are fundamentally different since all belong to the class of bioactive substances called allohormones (Koene and Ter Maat, 2001, 2002; Koene, 2004, 2005). Effects of the accessory gland products present in the ejaculate of snails have been investigated in most detail in *L. stagnalis*, which produces its seminal fluid in the prostate gland (reviewed in Jarne et al., 2010; Koene, 2010, 2016). This species’ prostate gland produces a number of different proteins and peptides that are transferred to the partner (Koene et al., 2010). One of these, referred to as Ovipostatin or LyAcp10, induces a delay in egg laying of recipients (Koene et al., 2010), resulting in a higher investment per egg as well as possibly enhancing storage duration, and/or fertilization chances of the donated sperm (Hoffer et al., 2012; Swart et al., unpublished). The latter outcome still needs to be tested, but is predicted because a delay in egg laying enables the donated sperm to reach the sperm storage site before a new set of eggs is fertilized and the egg mass is laid. The delay may also favor larger eggs because the resources available in the albumen gland, which provisions the eggs with galactogen‐rich perivitellin fluid (Jarne et al., 2010; Koene, 2010), keep accumulating (Koene and Ter Maat, 2004; Van Iersel et al., 2014; Swart et al., unpublished). Of course, components within the egg can also affect offspring development, as was recently shown to be the case for the amount of serotonin present in this species’ eggs (Ivashkin et al., 2015). Exploring whether components added to the egg are also influenced by accessory gland products will be an interesting next step.

Ovipostatin could serve as a cue for the female reproductive system to delay egg laying because the received ejaculate needs to be processed, thus coordinating the recipient's reproductive processes (see also Koene, 2016). Alternatively, it could act as a manipulative agent that “hijacks” receptors normally used for the regulation of egg laying, thus being beneficial for the donor. To fully understand how this delayed egg laying effect is mediated by Ovipostatin, it will be instructive to investigate if this peptide affects the excitability of the main neuroendocrine center that controls egg laying, the bilateral caudo‐dorsal cell cluster in the cerebral ganglia (e.g., Ter Maat et al., 1983), or if it works via a different route.

Recent work has also revealed that two identified accessory gland proteins, LyAcp5 and LyAcp8b, affect the male function of the recipient in addition to the egg laying process (Nakadera et al., 2014). The male‐function effect appears to be unique for simultaneous hermaphrodites: these proteins cause a recipient snail to transfer half the amount of sperm to its next partner. This sperm‐number‐reduction effect has been demonstrated both after natural insemination and artificial injection (via the female gonopore) with the isolated protein (Nakadera et al., 2014). Moreover, this reduction in sperm numbers is relevant for paternity success because such snails achieved less paternity with their recipient (Nakadera et al., 2014). Therefore, these hermaphrodites can directly influence their partner's male reproductive success (called a “cross‐sex effect”) (Anthes et al., 2010) in addition to its female physiology and investment. Interestingly, no evidence was found for a decreased motivation to inseminate a partner after receipt of an ejaculate (Nakadera et al., 2015), even though this seems a likely behavioral response to the receipt of these sperm‐number‐reducing peptides. The only phenomenon in molluscs that might achieve a similar reduction in investment in the male function is apophallation, where the penis of the partner is bitten off and thus male mating is eliminated (Leonard et al., 2002; but see Reise, 2007)—although clear evidence for this is still lacking.

Long‐term experiments in *L. stagnalis* revealed that higher male mating success results in higher male reproductive success, but also that higher female mating success negatively affects the individual's male reproductive success (Hoffer et al., unpublished). The latter observation is explained by the negative effect these accessory gland products have on the sperm transfer of the recipient (Nakadera et al., 2014), thus providing novel insight into post‐copulatory sexual selection in a separate‐sexed context. Clearly, these effects of accessory gland products need to be further investigated mechanistically (see Koene, 2016) and verified in other hermaphroditic snails. Moreover, these behavioral outcomes deserve attention in separate‐sexed species, particularly by identifying situations where males can potentially suppress the reproductive success of rivals prior to sperm transfer (Zizzari et al., unpublished). In this respect, it is very interesting to note that sequences matching Ovipostatin have recently been found in the *B. glabrata* genome (Adema et al., unpublished), suggesting that the observed reduction in egg laying under the influence of mating in this mollusc may be driven by Ovipostatin‐like regulation (Swart et al., unpublished). In contrast, none of the other male accessory gland proteins identified in *L. stagnalis* were found in the *B. glabrata* genome (Adema et al., unpublished), nor do they match with other known proteins (Koene et al., 2010), indicating that these accessory gland proteins probably evolve rapidly, and are putatively taxon‐specific.

## SHIFTING SEX ALLOCATION

**Figure 2:**
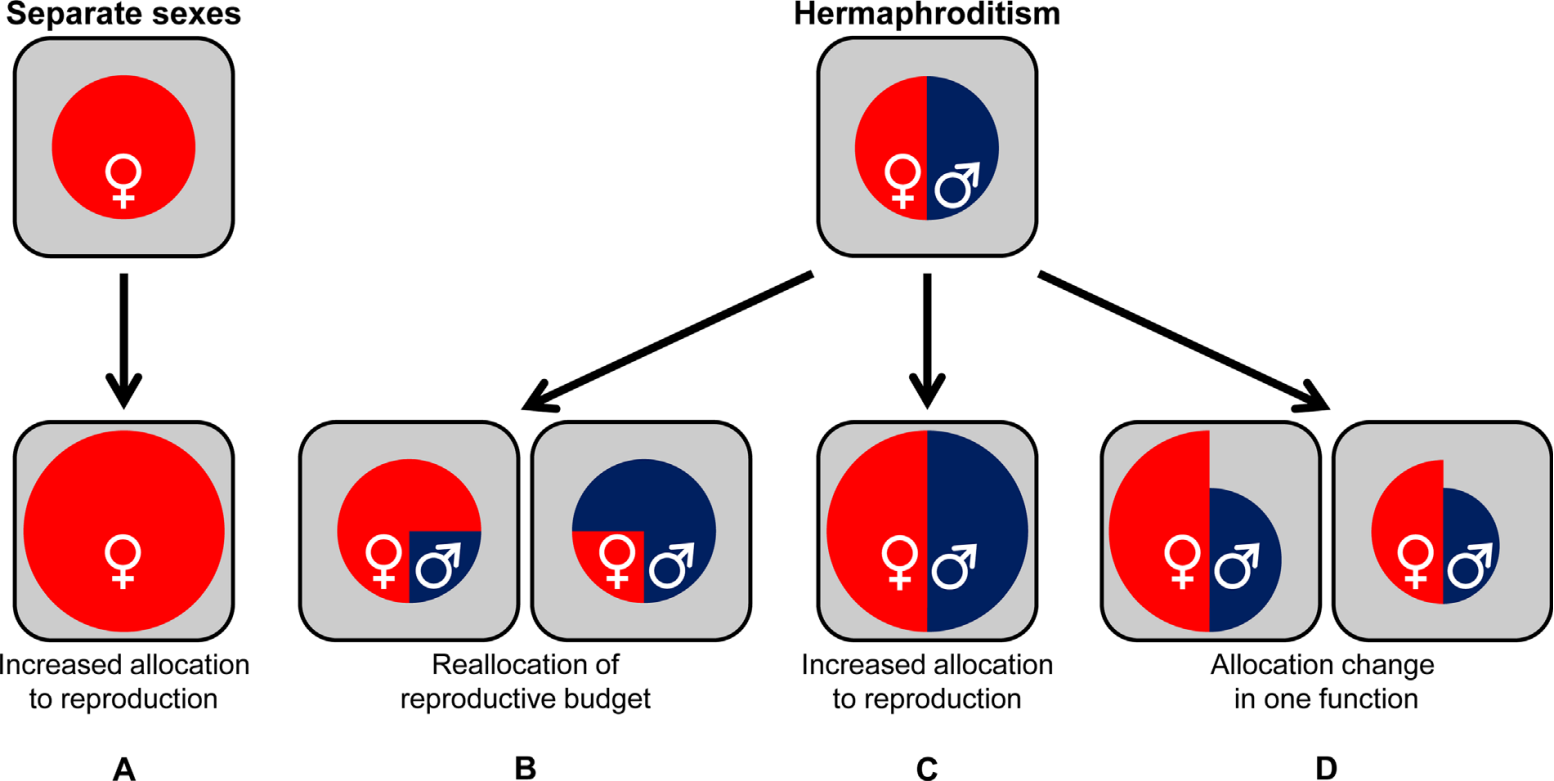
Changes in reproductive allocation due to accessory gland proteins. **A**: In species with separate sexes, overall investment in reproduction can be increased at the expense of the non‐reproductive energy budget (indicated by the grey area). **B**: In simultaneous hermaphrodites, in line with the general assumption of sex allocation theory, allocation can be shifted in either direction without affecting the non‐reproductive budget. In this case, female investment is most likely to increase (left option). **C**: As is separate‐sexed species, the overall reproductive investment can be increased. **D**: Alternatively, the investment in one specific sexual function can be changed without sacrificing investment in the other sexual function but rather investment in the nonreproductive budget; an increase most likely occurs in the female function (left option), a decrease is more likely for the male function (right option).

As illustrated with the above examples, the investment in male and/or female reproduction can be influenced both by mating history and accessory gland protein receipt. Moreover, sex allocation can be affected in different ways—some of which are impossible in species with separate sexes (Fig. 2) (see also Schärer and Ramm, 2016). For both sexual systems, investment in reproduction can simply be increased, taking resources away from the overall, non‐reproductive energy budget, resulting in increased reproduction at the expense of other somatic/maintenance functions—the most obvious being growth and survival (e.g., Rice, 1996) (Fig. 2). When this is mediated by male accessory gland proteins, this phenomenon is generally interpreted as sexual conflict, but it remains possible that some accessory gland proteins are instead used as signals or cues for (cryptic) mate choice, as in when to increase investment in offspring. This shift in reproductive investment is comparable between separate sexed organisms, where female investment is increased (Fig. 2A), and simultaneous hermaphrodites, where male as well as female investment are increased (Fig. 2C).

Such an overall increase in reproduction in hermaphrodites may not seem unlikely, but goes against the idea that hermaphrodites have evolved to be flexible in dividing their resources over their sexual functions. One perspective is that simultaneous hermaphrodites have a fixed reproductive budget to optimally divide over the two sexual functions, but it should be noted that this general assumption is a simplified model (e.g., Charnov, 1979; Schärer, 2009). Irrespective of whether or not this budget is fixed—for which we currently have no empirical evidence—increased investment in one sexual function may be at the expense of the other sexual function (Fig. 2B). The logical options, from the sperm donor's perspective, are an increase in female investment or an increase in the chances to fertilize eggs (left option of Fig. 2B). If one lets go of this fixed‐reproductive‐budget assumption, another option is a change in only one of the sexual functions, where an increase in the female function seems most logical (left option of Fig. 2D).

The above clearly highlights that one cannot fully distinguish between these different options with the currently available data. For example, despite the clear paternity benefit for investing in the love dart from the male perspective, there is very limited evidence for this being costly for the recipient (Kimura and Chiba, 2015; Lodi and Koene, 2016a). Likewise, the reduction in sperm transfer after insemination that is observed in the pond snail *L. stagnalis* does not seem to go along with a parallel increase in egg laying (Nakadera et al., 2014; Schärer et al., 2014). Reduced sperm transfer would be explained if overall male allocation is relatively small (i.e., much less than half) or if the ejaculate itself is not the most costly component of male reproduction; the latter is predicted to be the case, given the observation that individuals restricted to perform the male role spend an equal amount of resources as female‐only individuals, using eggs investment as a currency (Hoffer et al., 2010). Determining if is correct, for any species, requires disentangling the costs that are allocated towards the performance of courtship and copulation from the energetic value of an ejaculate containing sperm and accessory gland protein; the same can be done for female allocation, which involves separating the investment in egg laying behavior from the actual investment in the egg mass. These costs can be more easily disentangled in a unilaterally mating species than in a reciprocally mating species since the sexual roles can be clearly separated experimentally during unilateral mating.

**Figure 3:**
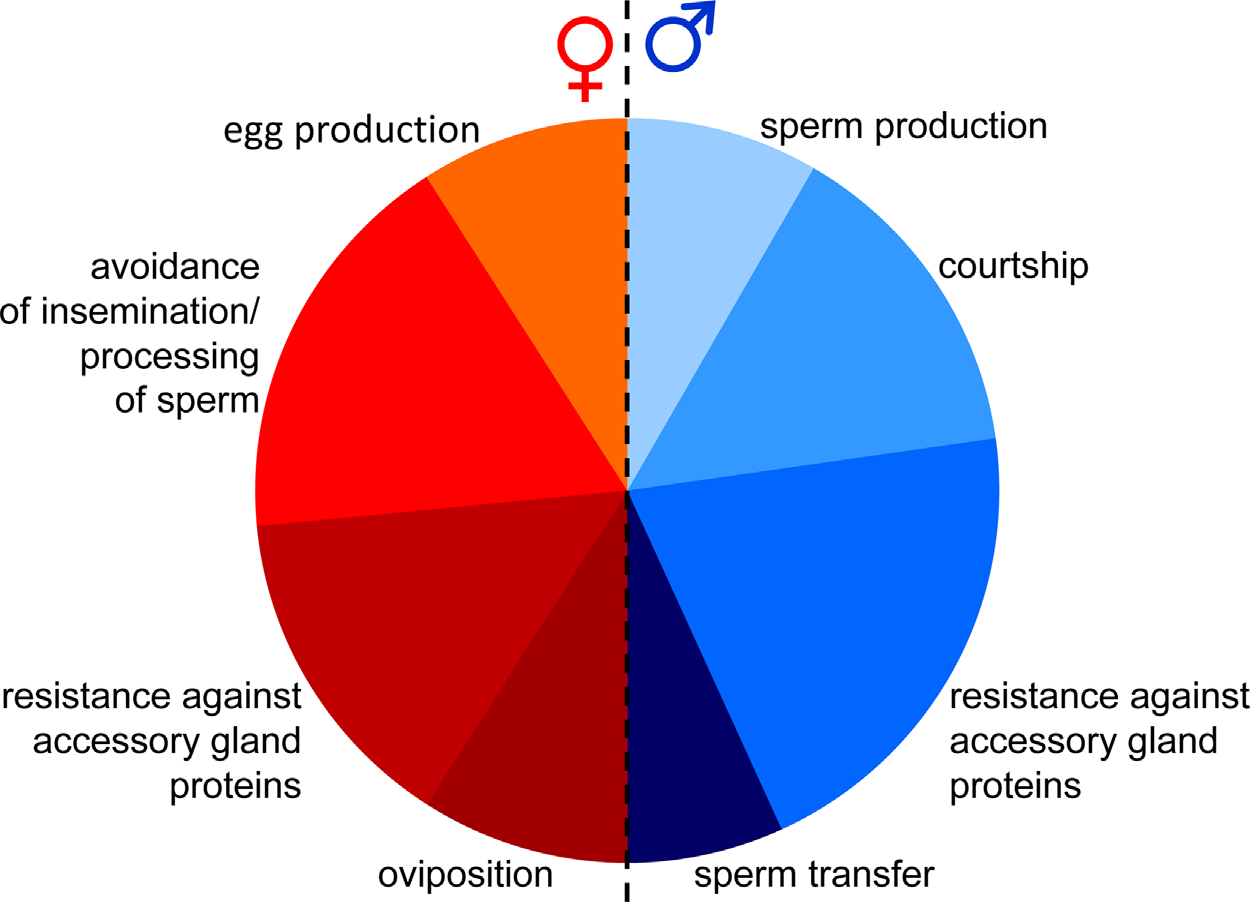
Schematic and hypothetical representation of how the different components of reproductive investment can contribute to the male and female function of a hermaphrodite. For the female function, energy is invested in egg production, avoidance of insemination and processing of sperm, resistance to accessory gland proteins, and oviposition. For the male function, energy is invested in sperm production, courtship, resistance to accessory gland proteins, and transfer of sperm. Note that this is specified for what is currently known about snails, but some components might also apply to the opposite sex (such as courtship by the female function, as occurs in other animal groups). In addition, one can imagine components that apply to both sexual functions, such as body size, which depend on nonreproductive investment but may partly be influenced by sexual selection.

Irrespective of where the costs lie, the division of resources over the functions of either sex can lead to sexual conflict in simultaneous hermaphrodites. The degrees of freedom for how this is expressed are larger, especially considering that either sex can develop resistance and/or counter‐adaptations to avoid inflicted costs (Figs. 2 and 3). One also has to keep in mind that the “what you give is what you get” principle might restrict the extremes that can be taken: For example, targeting accessory gland proteins to an existing reproductive pathway, which can develop resistance to such accessory gland proteins, might actually undermine the efficiency of hermaphroditic reproduction via an insensitivity to one's own regulatory substances (Koene, 2005). Along the same lines, the donation of a beneficial substance to the partner in the form of a nuptial gift is unlikely, given that such a gift could end up being exchanged, leading to no net gain nor guarantee of it being invested into egg production (see also Lewis et al., 2014; Schärer et al., 2014; Lodi and Koene, unpublished).

A second conclusion that can be drawn is that the investment is intertwined with motivation, especially when the performance of the sexual roles itself is energetically costly. This is clearly in agreement with the gender‐ratio hypothesis (Anthes et al., 2006), which assumes that relative fitness payoffs for each sexual role, and thus the preferred mating roles, vary and may switch with different partners. Hence, the motivation to perform a specific sex role is flexible, depending on the potential gains in male versus female fitness. This also highlights how detection of the potential partner's condition, health, and quality as metrics of fitness need to be investigated in detail in hermaphrodites (Koene, 2016).

Finally, although much is known in cases with a clear distinction between the sexes, for a more complete understanding of reproductive investment in relation to sex determination these processes really need to be investigated and critically analysed in other sexual systems (asexual, parthenogenetic, selfing, and simultaneous and sequential hermaphroditic). In other words, as already pointed out by Charnov (1982), think of the flexible allocation of resources to the two sexes within hermaphrodites as a more quantitative decision about the allocation of resources (reviewed by Yusa, 2007; Schärer, 2009; Beukeboom and Perrin, 2014). The great advantage of using this perspective to contemplate sex determination and sex ratio is that mechanisms across different types of sexual systems become equalized and are thus more comparable. The Gastropoda is indeed a useful source for such comparison since this taxonomic class displays all types of sexual systems (see Auld and Jarne, 2016). Using such an approach will foster the exploration of commonalities and differences between different sexual systems, thus helping determine whether sex determination is similar to or different from gender expression and sex allocation—or if the phenotype tracks along a sliding scale that can become genetically fixed—as well as defining any mechanistic differences in regulation between different sexual systems.
